# Peri- and Postanalgesic Properties of Lidokain, Lornoxicam, and Nitroglycerine Combination at Intravenous Regional Anesthesia

**DOI:** 10.1155/2014/737109

**Published:** 2014-03-09

**Authors:** Biricik Melis Cakmak, Gokhan Cakmak, Elif Akpek, Gulnaz Arslan, Mehmet Sukru Sahin

**Affiliations:** ^1^Anesthesiology and Reanimation Department, Baskent University School of Medicine, Baskent University, Alanya Hospital, Saray Mah, Kızlarpınarı Caddesi No. 1, 07400 Alanya, Antalya, Turkey; ^2^Orthopedics and Traumatology Department, Baskent University School of Medicine, Baskent University, Alanya Hospital, Saray Mah, Kızlarpınarı Caddesi No. 1, 07400 Alanya, Antalya, Turkey; ^3^Anesthesiology and Reanimation Department, Baskent University School of Medicine, Baskent University, Ankara Hospital, Fevzi Çakmak Caddesi No. 10 Sokak No. 45, Bahçelievler, 06490 Ankara, Turkey

## Abstract

*Background.* This study was conducted to compare and evaluate the effect of adding lornoxicam or nitroglycerine as adjuncts to lidocaine in intravenous regional anesthesia (IVRA). *Methods.* 60 patients were randomly separated into three groups, lidocaine group (group L), lidocaine + lornoxicam group (group LL), and lidocaine + lornoxicam + transdermal nitroglycerine group (group LL-N). Hemodynamic parameters, sensory and motor blocks onset, and recovery times were recorded. Analgesic consumption for tourniquet pain and postoperative period were recorded. *Results.* Sensory block onset times and motor block onset times were shorter in the LL-N and LL groups compared with L group. Sensory block recovery time and motor block recovery time were prolonged in the LL and LL-N groups compared with group L. The amount of fentanyl required for tourniquet pain was less in group LL and group LL-N when compared with group L. VAS scores of tourniquet pain were higher in group L compared with the other study groups. Postoperative VAS scores were higher for the first 4 hours in group L compared with the other study groups. *Conclusion.* The adjuvant drugs (lornoxicam or TNG) when added to lidocaine in IVRA were effective in improving the overall quality of anesthesia, reducing tourniquet pain, increasing tourniquet tolerance, and improving the postoperative analgesia.

## 1. Introduction

Intravenous regional anesthesia (IVRA) is widely recommended and applied in patients undergoing ambulatory procedures. Various additives have been used with local anesthetic agents to improve block quality, reduce tourniquet pain, and prolong postdeflation analgesia [1]. The potential intraoperative benefit ofnonsteroidal anti-inflammatory drugs (NSAIDS) added to local anesthetic agents have been demonstrated at several studies [2, 3]. Lornoxicam is a new NSAID of the oxicam class which is available in oral and parenteral forms. It is a nonopioid analgesic as effective as morphine, tramadol, and meperidine and produces less adverse effects than others [4]. Transdermal nitroglycerine (TNG), nitric oxide generator, helps in distribution of local anesthetic agents to neuron trunks by vasodilatation and also it has been demonstrated that, when transdermal nitroglycerine is used with other drugs, analgesic effect is increased. Nitric oxide and NSAI drug combinations are produced. It is called (NONSAID) for reducing adverse effects of NSAI drugs which are caused by COX enzyme inhibition [5–9]. This study was designed to compare and evaluate the effect of adding lornoxicam or both lornoxicam and TNG as adjuncts to lidocaine for IVRA.

## 2. Methods

With hospital ethics committee approval and informed written consent, we recruited 60 nonpremedicated ASA physical status I-II 18- to 40-year-old patients undergoing elective hand, wrist, and forearm surgery procedures. Patients with Raynaud's disease, sickle cell anemia, and history of drug allergies were excluded from the study.

The study design was randomized and double-blinded. An anesthesia assistant who was blinded to study prepared identical syringes containing each drug according to randomisation list. At premedication room, two cannulae were placed; 22-gauge cannula was placed in dorsal vein of operative hand for applying study drugs; and other cannulae was placed in the opposite hand for fluid 5% ringer lactate infusion administration.

Monitoring includes measurement of arterial blood pressure (mean arterial pressure (MAP)), heart rate (HR), and saturation of peripheral oxygen (SpO_2_) (Taema Artema MM206, Artema Medical AB Sundbyberg, Sweden). After application of standard monitoring, operative arm was elevated for two minutes and was then exsanguinated with an Esmarch bandage from distal to proximal. We placed a pneumatic tourniquet which has double cuff around the upper arm. Proximal cuff was inflated to 250 mmHg, and distal tourniquet was not inflated. Circulatory isolation of the arm was verified by inspection, absence of radial pulse, and loss of pulse oximetry racing in the ipsilateral index finger. The tourniquet period was from the time at which the distal cuff was inflated to the time the patient experienced pain.

The syringes contained 3 mg/kg lidocaine 2% (Aritmal, TEMS, Istanbul, Turkey) diluted with saline to a total volume of 40 mL in all groups for IVRA.

Patients were randomized to three groups with 20 patients in each.

*Group I (Group L) (Lidocaine Group).* 3 mg/kg lidocaine 2% (Aritmal, TEMS, Istanbul, Turkey) was diluted with saline to a total volume of 40 mL. Two hours before the operation, empty TNG flaster (Nitroderm, TTS flaster, Novartis) was placed on the hand at which the operation would be performed.

*Group II (Group LL) (Lidocaine *+* Lornoxicam Group).* 3 mg/kg lidocaine 2% (Aritmal, TEMS, Istanbul, Turkey) was diluted with saline to a total volume of 40 mL and also 8 mg lornoxicam (Xefo, Abdi Ibrahim, Turkey) was added to the solution. Two hours before the operation, empty TNG flaster (Nitroderm, TTS flaster, Novartis) was placed at the hand at which the operation would be performed.

*Group III (Group LL-N) (Lidocaine + Lornoxicam *+* TNG Group).* 3 mg/kg lidocaine 2% (Aritmal, TEMS, Istanbul, Turkey) was diluted with saline to a total volume of 40 mL and also 8 mg lornoxicam (Xefo, Abdi Ibrahım, Turkey) was added to the solution. Two hours before the operation, TNG flaster which contains 5 mg nitroglycerine (Nitroderm, TTS flaster, Novartis) was placed at the surgical site.

The study solutions were injected over 90 seconds by an anesthesiologist blinded to the study drugs.

After IVRA was achieved, sensory block was assessed by a pinprick testing performed with a 22-gauge short-beveled needle. Patient response was evaluated in the dermatomal sensory distribution of ulnar, median, and radial nerves. Sensory block onset time was noted as the time elapsed from injection of study drug to sensory block achieved in all dermatomes.

Motor function was assessed by asking the subjects to flex and extend his/her wrist and fingers, and complete motor block was noted when no voluntary movement was possible. Motor block onset time was the time elapsed from injection of the study drug to complete motor block.

After sensorial and motor blocks were assessed at all dermatomes, distal cuff was inflated to 250 mmHg, and proximal cuff was deflated after taking out TNG patch. Pain due to the tourniquet was assessed with a 10 cm visual analogue scale (VAS) (0 = no pain and 10 cm = worst pain imaginable). HR, MAP, SPO2, and VAS were monitored and recorded before and after the application of the tourniquet and 1, 5, 15, 30, and 45 minutes after the injection of local anesthetic solution.

If the patient reported VAS > 3 during the surgery, 1 *μ*g/kg IV fentanyl (fentanyl citrate; Abbott, North Chicago, IL) was given. Total fentanyl requirement (dose and time) was recorded.

During the surgery time, 4 mg IV ondansetron hydrochloride (Zofran, GlaxoSmith Kline) was given for nausea and vomiting, 5 mg IV ephedrine was given for hypotension (systolic arterial blood pressure < 90 mmHg or 50 mmHg lower than the normal value), and 0.5 mg IV atropine was given for bradycardia (HR < 50/min). All of these complications were also recorded with respect to time.

After the surgery, the anesthesiologist, who did not know what medication was given by TNG patch and injection, was asked to qualify the anesthetic conditions according to the following numeric scale.

At the end of the operation the patients were asked to qualify the operative conditions such as tourniquet pain or incisional pain according to the following numeric scale.

 Excellent (4) = no complaint from pain.
 Good (3) = minor complaint with no need for supplemental analgesics.
 Moderate (2) = complaint which required supplemental analgesic.
 Unsuccessful (1) = patient given general anesthesia.

At the end of the surgery, the surgeon, who was blind to patient group, was asked to score operative conditions such as disturbing movement of arm and excessive bleeding according to the following numeric scale [10]:

 0 = unsuccessful;
 1 = poor;
 2 = acceptable;
 3 = good;
 4 = excellent.

The tourniquet was not deflated before 30 minutes and was not inflated more than 2 hours. At the end of surgery, the tourniquet deflation was performed by the cyclic deflation technique. After tourniquet deflation, sensory recovery time (the time elapsed after tourniquet deflation up to recovery of pain in all innervated areas determined by pinprick test done every 30 seconds) was noted. Motor block recovery time (the time elapsed after tourniquet deflation up to movement of fingers) was noted. First analgesic requirement time (the time elapsed after tourniquet release to the first patient request of analgesic) was also noted.

Nausea, vomiting, skin reactions, dizziness, tinnitus, tachycardia, bradycardia, hypotension, and hypertension were noted until discharge from the recovery room and at the end of the 24 h postoperative observation period.

IM diclofenac (Voltaren; Ciba-Geigy, İstanbul, Turkey) 75 mg was given to patients with a VAS > 3 at postoperative first 8 hours and total diclofenac requirements (time and amount) were recorded by a blinded anesthesia resident. Patients were instructed to take one peroral paracetamol (Parol tablet 500 mg; Atabay) tablet at postoperative 8–24 hours required for a VAS > 3 while at home. All the patients were called by telephone the day after surgery by a blinded observer. The time from tourniquet deflation until the patient first required analgesic, diclofenac im or/and peroral paracetamol, was noted at the first postoperative 24-hour period.

### 2.1. Statistical Analysis

The statistical evaluation was done using SPSS 17.0 for Eindows (SPSS Inc., Chicago, IL, USA). Initial sample size estimation showed that approximately 18 patients were needed in each group to detect a clinically relevant reduction of fentanyl consumption by 25% and also approximately 50% clinically significant changes of the sensory block onset and recovery times with a power of 80% and a level of significance of 5%. Independent samples *t*-test was used for evaluation of the demographic data, hemodynamic data, the time of sensory and motor block onset and the recovery time, duration of surgery and tourniquet, initial time of tourniquet pain, VAS scores, postoperative first analgesic requirement time, and intraoperative and postoperative analgesic use. Mann-Whitney *U* test was used for intraoperative and postoperative quality of anesthesia. Level of significance was determined at *P* > 0.05 for no statistically significant difference and at *P* < 0.05 for significant difference.

## 3. Results

Demographic data of the groups were similar to mean age, weight, height, and sex ratio (Table 1). All patients were able to complete the study, and there were no exclusions in data analysis. There were no statistical differences between groups' duration of surgery and tourniquet time (Table 1). Sensory block onset times were shorter in the LL-N (3.4 ± 0.31 minutes) and LL (5.10 ± 0.38 minutes) groups compared with group L (6.2 ± 0.33 minute) (*P* < 0.001) and motor block onset times were shorter in the group LL (8.4 ± 1.6 minutes) and group LL-N (4.7 ± 1.2 minutes) compared with group L (11.2 ± 1.5 minutes) (*P* < 0.0001). Sensory block recovery time was prolonged in the LL (7.6 ± 0.72 minutes) and LL-N (8.9 ± 0.77 minutes) groups compared with group L (3.1 ± 0.53 minutes) (*P* = 0.001). Motor block recovery time was prolonged in the LL (8.4 ± 1.4 minutes) and LL-N (7.9 ± 1.2 minutes) groups compared with group L (7.1 ± 1.2 minutes) (*P* = 0.0014 and 0.023, resp.) (Tables 2 and 3). The number of patients who need additional fentanyl requirements were significantly more in group L than the other two groups (*P* = 0.047), but amount of fentanyl required for tourniquet pain was statistically similar in all groups (Table 4). There was also no statistical difference among groups when compared for MAP, HR, and SPO2 at any time either intraoperative or postoperative time (data are presented in Table 5) (*P* > 0.05).

At the first 8 hours of postoperative period, the number of patients who need additional diclofenac and diclofenac consumption was statistically not different between the groups.

Both the number of patients who need additional paracetamol and paracetamol amount which was used at postoperative (8–24 h) period were statistically more at group L than the other two groups. There was significant difference in paracetamol requirement between the groups L and LL-N (*P* = 0.001).

No patient suffered from incisional pain during intraoperative period in all groups. VAS scores of tourniquet pain were higher at 10, 20, 30, and 40 minutes in group L compared with the other study groups (*P* < 0.0001). Postoperative VAS scores were higher for the first 4 h in group L compared with the other study groups (*P* < 0.0001) (Table 6). Anesthesia quality, determined by anesthesiologist and the patient, was better in groups LL and LL-N than in group L (*P* < 0.0001) (Table 7). There was not any significant difference in side effects between the groups (*P* > 0.05).

## 4. Discussion

The main result of our study revealed that the addition of both lornoxicam and nitroglycerin during IVRA improved speed of sensory and motor block onset times, decreased tourniquet pain, improved quality of anesthesia, and decreased intraoperative and postoperative analgesic consumption without causing any side effects.

Intravenous regional anesthesia (IVRA) is a simple, common, and a reliable method which provides adequate anesthesia and muscle relaxation at short operative procedures of extremity surgery. Lidocaine is the most commonly chosen local anesthetic for IVRA. But, there are well-known limitations to this local anesthetic especially at prolonged surgery and postoperative period.

Various additives have been used with local anesthetics to improve block quality, reduce tourniquet pain, and prolong postdeflation analgesia and varying results with the possibility of additional complications have occurred. Additives used were opioids (fentanyl, meperidine, morphine, and sufentanil), tramadol, NSAIDs (ketorolac, tenoxicam, lornoxicam, and acetylsalicylate), clonidine, dexmedetomidine, nitroglycerine, muscle relaxants (atracurium, pancuronium, and mivacurium), alkalization with sodium bicarbonate and potassium [1, 10–14].

NSAIDs inhibit the production of prostaglandins from arachidonic acid in phospholipid membranes. The result is decreased afferent nociceptive signal arising from the site of surgery and also they act at peripheral nociceptors, perhaps by interfering with the synthesis and activity of pain mediators derived from arachidonic acid, and can supplement postoperative pain relief. NSAIDs as a part of IVRA have longer analgesic benefit than the same dose parenterally administered [1, 15].

Lornoxicam (chlortenoxicam) is a nonselective NSAID of the oxicam class, with analgesic, anti-inflammatory, and antipyretic effects. It is a highly potent short acting analgesic agent. It is available in oral and parenteral forms. It is separated from established oxicams by a relatively short elimination half-life (3 to 5 hours); this may be suggested as advantageous for use in postoperative period and also advantageous due to tolerability. In particular, it has a tolerability profile characteristics of NSAIDs, with gastrointestinal disturbances (pain, dyspepsia, nausea, and vomiting) being the most remarkable events. Lornoxicam is highly effective in both relieving postoperative pain and reducing the need for rescue analgesics following different surgical procedures [4, 15–23]. The beneficial effect of lornoxicam on postoperative pain relief in our study was clinically evident by lower pain scores and longer time to diclofenac rescue request with a reduction in the first 8- and 24-hour analgesic consumption, but it is not statistically evident [19].

Sen and colleagues show that addition of NSAID (lornoxicam) to lidocaine for IVRA shortens the onset of sensory and motor block, decreases tourniquet pain, and improves postoperative analgesia without causing any side effects. In another study, they add NSAID (ketorolac) to lidocaine for IVRA and they conclude that ketorolac improves IVRA with lidocaine in terms of controlling intraoperative tourniquet pain by diminishing postoperative pain [16]. NSAIDs as a part of IVRA have longer analgesic benefit than the same dose parenterally administered [1]. We have drawn a similar conclusion in our study both shorter sensory and motor block onset times and longer sensory and motor recovery times that received lidocaine plus lornoxicam than only lidocaine group. This result can be explained by the effect of alkalization. Alkalization with bicarbonate as an adjunct for IVRA is used widely. By alkalization, it is possible to increase the amount of free base LA; this helps in the nerve penetration easily and onset of blockade faster. The pH of lornoxicam intravenous form is approximately 8.7, the pH of the lidocaine solution is 6.7, and the pH of lornoxicam-lidocaine mixture is 7.6 [17].

NTG shows its analgesic effect as it is metabolized to nitric oxide (NO). NO causes an increase, in the intracellular concentration of cyclic guanosine monophosphate (c-GMP), which produces pain modulation in the central and peripheral nervous system [5, 24, 25]. The injected LA diffuses into the small veins surrounding the nerves and then into the vasa nervorum and capillary plexus of the nerves, leading to a core-to-mantle (centrifugal) conduction block in the nerves involved. It then spreads around the small nerves in the skin, blocking their conduction. Several studies on NTG have demonstrated its analgesic effect in acute and chronic pain conditions. Berrazueta et al. proved the analgesic action of transdermal glycerylnitrate in the treatment of shoulder pain [8]. Lauretti et al. documented that transdermal NTG enhanced the postoperative analgesic effect of spinal sufentanil and neostigmine [7]. Also NTG has been studied as an adjuvant to IVRA. Abbasivash et al. studied the effect of adding NTG to lidocaine in IVRA where it shortened the onset times of sensory and motor block and decreased the tourniquet and postoperative pains, without any side effect [26]. Sen et al. studied the addition of NTG to lidocaine in IVRA where it shortened sensory and motor block onset times, prolonged sensory and motor block recovery times, and improved tourniquet pain while prolonging the time for the first analgesic requirement and decreasing the total amount of postoperative analgesic requirement without side effects [15]. Turan et al. suggested that transdermal NTG has useful effects on sensory and motor block without side effects in IVRA [27]. Our results seem to be similar to those of Abbasivashi et al. and Sen et al. Sensory and motor block onset times were statistically shorter in group LL-N than in group LL and group L. This could be explained by direct vasodilator effect of nitroglycerine that promotes distribution of lidocaine to nerves. There were also lower VAS scores for tourniquet pain and reduced paracetamol requirement time in group NTG. In our study, there was no significant difference in side effects between the three groups; this can be due to the fact that nitroglycerin produces antioxidative effect [27]. In that respect, the antioxidative effects of the drug might be particularly important for preventing gastrointestinal side effects. Transdermal nitroglycerin patch may, in fact, reduce gastric damage induced by parenteral administration of indomethacin [28]. It was also demonstrated that nitric oxide-releasing NSAIDs (NONSAID) can prevent gastrointestinal side effects in acute and chronic administration in animals. Sen et al. suggests that lornoxicam may cause gastrointestinal and renal side effects. Adding nitroglycerin to lornoxicam might prevent gastrointestinal and renal side effects compared to lornoxicam alone [16].

In conclusion, addition of lornoxicam or TNG to lidocaine in IVRA was effective in improving the overall quality of anesthesia, reducing tourniquet pain, increasing tourniquet tolerance, and improving the postoperative analgesia. The combination of lidocaine, lornoxicam, and TNG as an adjuvant produced faster onset of sensory and motor blockades in comparison to other groups. The underlying mechanisms are yet to be elucidated with more experimental studies.

## Figures and Tables

**Table 1:** Patients demographics data, duration of tourniquet, and surgery time.

	Group L (*n* = 20)	Group LL (*n* = 20)	Group LL-N (*n* = 20)	p1	p2	p3
Age (years)	47.70 ± 3.26	55.70 ± 2.38	51.30 ± 3.17	0.063	0.43	0.28
Gender (M/F)	14/16	16/14	17/13	0.67	0.66	0.38
Weight (kg)	76.20 ± 1.96	76.70 ± 2.4	73.20 ± 3.12	0.88	0.42	0.401
Height (cm)	161 ± 7	162 ± 6	161 ± 5	0.13	0.15	0.23
Tourniquet time (min)	43.00 ± 4.73	39.30 ± 3.51	39.50 ± 3.86	0.53	0.57	0.97
Surgery time (min)	35.10 ± 4.00	33.80 ± 3.60	34.50 ± 3.58	0.81	0.91	0.89

(Values are mean ± SD); p1: comparison of groups L and LL; p2: comparison of groups L and LL-N; and p3: comparison of groups LL and LL-N.

**Table 2:** Onset and recovery times of sensory and motor blocks (min).

	Group L (*n* = 20)	Group LL (*n* = 20)	Group LL-N (*n* = 20)	p1	p2	p3
Sensory block onset time (min)	6.20 ± 0.33	5.10 ± 0.38	3.40 ± 0.31	0.041	0.001	0.003
Sensory block recovery time (min)	3.10 ± 0.53	7.60 ± 0.72	8.90 ± 0.77	0.001	0.001	0.23
Motor block on set time (min)	11.3 ± 1.5	8.4 ± 1.6	4.7 ± 0.82	0.67	0.25	0.15
Motor block recovery time (min)	7.1 ± 1.2	8.4 ± 1.4	7.9 ± 1.2	0.001	0.001	0.1

(Values are mean ± SD); p1: comparison of groups L and LL; p2: comparison of groups L and LL-N; and p3: comparison of groups LL and LL-N.

**Table 3:** Initial time of tourniquet pain (min).

	Group L (*n* = 20)	Group LL (*n* = 20)	Group LL-N (*n* = 20)	p1	p2	p3
Initial time of tourniquet pain (min)	15.80 ± 5.41	7.00 ± 4.90	3.00 ± 3.00	0.24	0.009	0.17

(Values are mean ± SD); p1: comparison of groups L and LL; p2: comparison of groups L and LL-N; and p3: comparison of groups LL and LL-N.

**Table 4:** Total amount of fentanyl, diclofenac, and paracetamol requirement (*μ*g).

	Group L (*n* = 20)	Group LL (*n* = 20)	Group LL-N (*n* = 20)	p1	p2	p3
Total amount of fentanyl requirement (*μ*g)	51 ± 61	15 ± 32	7 ± 22	0.047	0.017	0.15
Diclofenac requirement (mg)—postoperative first 8 hour	60 ± 31	37 ± 39	45 ± 38	0.11	0.35	0.67
Paracetamol requirement (mg)—postoperative 24 hours	850 ± 579	450 ± 497	100 ± 210	0.17	0.001	0.55

(Values are mean ± SD); p1: comparison of groups L and LL; p2: comparison of groups L and LL-N; and p3: comparison of groups LL and LL-N.

**Table 5:** Distribution of heart rate (HR) values (beat/min) and mean arterial pressure (MAP) (mmHg).

	Group L (*n* = 20)		Group LL (*n* = 20)		Group LL-N (*n* = 20)		p1		p2		p3	
	HR (beat/min)	MAP (mmHg)	HR (beat/min)	MAP (mmHg)	HR (beat/min)	MAP (mmHg)	HR (beat/min)	MAP (mmHg)	HR (beat/min)	MAP (mmHg)	HR (beat/min)	MAP (mmHg)
Preop	72.80 ± 3.09	94.20 ± 6.07	72.50 ± 1.75	99.70 ± 6.36	71.00 ± 2.59	86.50 ± 6.14	0.93	0.54	0.77	0.38	0.77	0.15
15 min	71.50 ± 3.58	88.90 ± 5.61	69.70 ± 1.64	96.30 ± 5.72	69.90 ± 2.61	83.90 ± 5.85	0.65	0.36	0.72	0.54	0.94	0.14
30 min	68.50 ± 3.18	88.20 ± 4.01	67.90 ± 1.55	97.70 ± 4.20	70.10 ± 2.77	77.30 ± 4.65	0.86	0.11	0.709	0.093	0.49	0.004
45 min	69.20 ± 2.56	88.80 ± 4.76	67.70 ± 1.01	97.30 ± 4.52	68.90 ± 2.93	85.40 ± 5.65	0.59	0.098	0.93	0.28	0.703	0.012

(Values are mean ± SD); p1: comparison of groups L and LL; p2: comparison of groups L and LL-N; and p3: comparison of groups LL and LL-N.

**Table 6:** Intraoperative tourniquet pain and postoperative VAS scores.

	Group L (*n* = 20)	Group LL (*n* = 20)	Group LL-N (*n* = 20)
5 minutes after tourniquet inflation	1 (0-1)	1 (0-1)	1 (0-1)
15 minutes after tourniquet inflation	1 (0-1)	1 (0-1)	1 (0-1)
30 minutes after tourniquet inflation	2 (0−3)	1 (0−2)	1 (0−3)
45 minutes after tourniquet inflation	3 (2−5)	2 (1−3)	3 (2−4)
5 minutes after tourniquet release	3 (2−5)	2 (2−5)	2 (2−4)
30 minutes after tourniquet release	4 (3−5)	3 (3−5)	3 (2−5)
2 h after tourniquet release	4 (3−5)	3 (3−5)	3 (2−5)

VAS scores of tourniquet pain were higher at 10, 20, 30, and 40 minutes in group L compared with the other study groups (*P* < 0.0001). Postoperative VAS scores were higher for the first 4 hours in group L compared with the other study groups (*P* < 0.0001).

**Table 7:** Quality of anesthesia assessed by anesthesiologists and surgeons.

	Group L (*n* = 20)	Group LL (*n* = 20)	Group LL-N (*n* = 20)
Quality of anesthesia (patient)	3 (2–4)	4 (3-4)*	4 (3-4)*
Quality of anesthesia (surgeon)	2 (2-3)	4 (3-4)*	3 (3-4)*

(Values are mean ± SD).

*P < 0.05 in comparison with control group.
